# The placenta protects the fetal circulation from anxiety-driven elevations in maternal serum levels of brain-derived neurotrophic factor

**DOI:** 10.1038/s41398-020-01176-8

**Published:** 2021-01-18

**Authors:** Hayley Dingsdale, Xinsheng Nan, Samantha M. Garay, Annett Mueller, Lorna A. Sumption, Pedro Chacón-Fernández, Isabel Martinez-Garay, Cedric Ghevaert, Yves-Alain Barde, Rosalind M. John

**Affiliations:** 1 School of Biosciences, Cardiff University, Cardiff, CF10 3AX UK; 2 Department of Haematology, University of Cambridge, Cambridge, CB2 0PT UK; 3 Present Address: Hospital Universitario Virgen Macarena-FISEVI, University of Seville, E41009 Seville, Spain

**Keywords:** Depression, Psychology

## Abstract

Brain-derived neurotrophic factor (BDNF) plays crucial roles in brain function. Numerous studies report alterations in BDNF levels in human serum in various neurological conditions, including mood disorders such as depression. However, little is known about BDNF levels in the blood during pregnancy. We asked whether maternal depression and/or anxiety during pregnancy were associated with altered serum BDNF levels in mothers (*n* = 251) and their new-born infants (*n* = 212). As prenatal exposure to maternal mood disorders significantly increases the risk of neurological conditions in later life, we also examined the possibility of placental BDNF transfer by developing a new mouse model. We found no association between maternal symptoms of depression and either maternal or infant cord blood serum BDNF. However, maternal symptoms of anxiety correlated with significantly raised maternal serum BDNF exclusively in mothers of boys (*r* = 0.281; *P* = 0.005; *n* = 99). Serum BDNF was significantly lower in male infants than female infants but neither correlated with maternal anxiety symptoms. Consistent with this observation, we found no evidence for BDNF transfer across the placenta. We conclude that the placenta protects the developing fetus from maternal changes in serum BDNF that could otherwise have adverse consequences for fetal development.

## Introduction

Perinatal mental health problems impact nearly one-quarter of all pregnancies^1^. In the UK alone, this costs society > £8 billion for each year of births^1^ due to the risk of substantial morbidity to mother and child^2^ and negative outcomes for the child^3–5^. Despite the considerable clinical and financial burden to society, the biological mechanisms linking maternal mood disorders to adverse outcomes are unknown.

One mechanism potentially accounting for the programming of disease risk is passage across the placenta of maternal factors impacting fetal development during sensitive periods, as has been suggested for the stress hormone cortisol^3,6^. Brain-derived neurotrophic factor (BDNF) is a member of the nerve growth factor family expressed in the central and peripheral nervous system with a key role in the development and function of neurons^7^. In primates, but not mice, significant levels of BDNF are detected in circulating blood platelets^8^, originating from megakaryocytes (MKs)^9^. Decreased serum BDNF has been reported in numerous conditions, including depression^10–15^, Huntington’s^16^ and Alzheimer’s disease^17^, whilst increased BDNF has been associated with autism spectrum disorder^18^. Circulating BDNF is also known to fluctuate with exercise^19^.

Pregnancy is a unique state where the blood systems of two individuals, the mother and the fetus, are intimately associated but not in direct contact. In humans, there is a barrier between the maternal and fetal circulations composed of a single continuous layer of syncytiotrophoblast over a layer of villous cytotrophoblast covering the endothelial cell-lined fetal blood vessels^20^. Despite significant morphological differences, rodents also possess the same hemochorial arrangement^21^, with the barrier consisting of a discontinuous layer of sinusoidal trophoblast giant cells and two continuous layers of syncytiotrophoblast covering fetal blood vessels^22^. Thus, in both cases, there are three cellular layers between maternal and fetal blood. Most proteins are too large to cross the placenta. One exception is maternal antibodies which cross the human syncytiotrophoblast by receptor-mediated pinocytosis, an active process requiring the neonatal Fc receptor^23^, and we note that a previous study in mice concluded that maternal BDNF may be another protein that can cross the placenta^24^.

Fluctuations in maternal serum BDNF may consequently adversely influence fetal development. A small number of studies have reported on maternal or fetal serum BDNF levels (see Supplementary Table 1^25–46^). Lower maternal BDNF has been associated with postnatal depression symptoms^46^ and lower cord blood BDNF associated with major depression in pregnancy^39^ but other studies report no association^33,37^. Two studies reported a relationship between cord blood serum BDNF and maternal anxiety symptoms, again with contrasting findings^33,42^. To date, no study has reported on the status of BDNF in blood from paired maternal and fetal samples in relation to maternal symptoms of both depression and anxiety.

In this study, we quantified BDNF levels in maternal serum and matched cord blood serum in relation to maternally reported symptoms of depression and anxiety, generated as part of the Grown in Wales study^47^. Mouse models have been extensively used to understand the function of brain-derived BDNF^48^; however, BDNF is not detectable in mouse MKs^9^ or blood^49^. Consequently, we genetically engineered BDNF expression from MKs to determine the potential for MK-derived maternal BDNF transfer across the placenta near term.

## Methods

### Grown in Wales study

GiW study is a longitudinal pregnancy cohort study. Women aged 18–45 with a singleton, term pregnancy without fetal abnormalities or infectious diseases were recruited at pre-surgical appointments prior to an elective caesarean section (ELCS) at the University Hospital of Wales. In total, 355 women provided written consent; 7 women subsequently withdrew. Perceived depression symptoms were assessed at recruitment using the Edinburgh Postnatal Depression Scale (EPDS)^50^ and trait anxiety by form Y-2 on the Spielberger State-Trait Anxiety Inventory (STAI) test^51^. Maternal venous serum samples were obtained at recruitment and 1–4 days later cord venous blood taken within 2 h of delivery. Blood was collected into Vacutainer blood collection tubes (BD Biosciences, 367837), inverted several times and incubated at room temperature (RT) for 1–2 h. After centrifugation at 3000 × *g* for 10 min at 4 °C, serum aliquots were stored at −80 °C. Prior to data analysis, samples were excluded based on the following pre-established *sample exclusion criteria*: women who went into natural labor prior to their planned ELCS were included in the analyses of maternal serum but their cord blood serum samples were excluded; 36 samples from <36 weeks^25^ and non-Caucasian^52^ pairs were also excluded.

### Serum BDNF ELISA

ELISA measurements of human serum were performed blind to all participant information except participant ID and type (maternal versus fetal), and as described^53^, with minor changes. Briefly, NeutrAvidin-coated plates (ThermoFisher Scientific, 15509) were washed with Buffer A (0.1% TritonX-100 in 0.1 M phosphate buffer: 0.1 M KH_2_PO_4_, 0.1 M Na_2_HPO_4_, pH 7.6) before incubating with 13 µg/ml biotin-BDNF-Ab#1^54^ for 2 h at RT. Plates were then washed with Buffer B (Buffer A with 1% BSA), before the addition of 150 µl Buffer A. Next, 50 µl of either sample (1:20 dilution in Buffer B) or standard (in Buffer B) was added and incubated for 6 h at RT while shaking. Following Buffer A washes, 1.25 µg/ml HRP-BDNF-Ab#9^54^ (in Buffer B) was added for 3 h at RT, again shaking. After further Buffer A washes, the chemiluminescent substrate (Roche, 11582950001) was added and signal detected by a microplate reader (FLUOstar OMEGA, BMG Labtech). All plates contained two repeated samples, and samples were analyzed in triplicate. ELISA measurements of mouse serum were performed as above, with the exceptions that experimenters were not blinded to sample information, and the sample incubation step was reduced to 3 h.

### Western blot analysis

Western blots were performed as described^53^. Proteins from 1 µl serum were separated on 4–12% Bis-Tris gradient gels (Invitrogen, NP0321BOX) and transferred to nitrocellulose membranes. After blocking for 1 h in 3% BSA (Sigma-Aldrich, A7906), 3% ECL Prime Blocking reagent (GE Healthcare) in TBS-T, membranes were incubated overnight with anti-BDNF 3C11 antibody (1:2000, Icosagen, 327-100). After washing, 1 h incubation with HRP-conjugated goat anti-mouse IgG1 (1:2000, Invitrogen, PA1-74421) followed, and membranes washed and developed.

### Generation of Rosa26-LSL-BDNF-myc-IG mice

The pZDonor-Mouse-Rosa26 plasmid (Sigma-Aldrich, D9196) was used as the backbone of a Rosa26 targeting vector. We inserted a 4185 bp PCR fragment, containing chimeric intron–exon-loxP-NeopA-loxP-MCS-IRES-eGFP-pA amplified from the pCAGfloxNeoIRESeGFP plasmid (gift from Professor Meng Li, Cardiff University) as a template, to the EcoRI/XmaI site to generate pZDR-LSL-IG. To generate pZDR-LSL-BDNF-myc-IG, a fragment containing the BDNF-myc coding sequence was isolated by double digestion with EcoRI/SacI from pCMV-BDNF-myc (constructed by adding one myc-tag copy at the C-terminus of WT mouse BDNF following the deletion of the last three amino acids^55^) and inserted into the PmeI site of pZDR-LSL-IG.

Mouse ES cells E14TG2a were cultured in gelatin-coated flasks or Petri dishes in GMEM medium (ThermoFisher Scientific, 11710035) with LIF (homemade) and 10% FCS (Biosera, FB-1001/500). To generate Rosa26-LSL-BDNF-myc-IG mouse ES cells, cells were transfected with pZDR-LSL-BDNF-myc-IG and pCMV-RosaL6 ELDmut and pCMV-RosaR4 KKRmut (gifts from Charles Gersbach, Addgene plasmid #37198 and #37199^56^) in a 10:1:1 ratio. Transfection was performed in Nucleofector solution P3 (Lonza, V4XP-3024) using 4D-Nucleofector X Unit (Lonza, AAF-1002X), program CG-104. 5′-homologous recombination was confirmed by generation of a 2.2 kb PCR fragment using primer pair (GTGGAGCCGTTCTGTGAGAC and GTCATAGCCGAATAGCCTCTCCAC), 3′ homologous recombination by a 1.3 kb PCR fragment using primer pair (TCGAGCTGGACGGCGACGTAAAC and TTGGGGGAGGAGACATCCACCTGG).

Rosa26-LSL-BDNF-myc-IG mice (BDNF-myc hereafter) were generated by injection of Rosa26-LSL-BDNF-myc-IG mouse ES cells into C57BL/6 blastocysts. Germline transmission was genotyped by PCR as above. Subsequent genotyping was conducted by PCR using two primer pairs (Bdnf pair: GAACTACCCAATCGTATGTTCG and CTACAAGTCTTCTTCAGAAATAAGCTT; Rosa26 pair: AAGGGAGCTGCAGTGGAGTA and GTCCCTCCAATTTTACACC) to identify heterozygotes (140 bp and 275 bp products), homozygotes (140 bp), and wild-type animals (275 bp).

### Generation of animals expressing transgene BDNF-myc in platelets

Pf4-Cre mice^57^ (The Jackson Laboratory, 008535, C57BL/6 background) were crossed with BDNF-myc animals to generate het BDNF-myc::Pf4-Cre mice. Homozygous BDNF-myc::Pf4-Cre mice were generated by crossing homozygous BDNF-myc with homozygous BDNF-myc::Pf4-Cre mice. Pf4-Cre genotyping was by the production of a 420 bp Pf4-Cre product (primer pair CCAAGTCCTACTGTTTCTCACTC and TGCACAGTCAGCAGGTT) and an internal 302 bp (Y chromosome) and/or 331 bp (X chromosome) (primer pair CTGAAGCTTTTGGCTTTGAG and CCACTGCCAAATTCTTTGG).

### Mouse megakaryocyte culture

Mouse megakaryocytes (Mks) were differentiated from bone marrow as described^9^. Briefly, bone marrow was flushed from femurs and tibias with Hank’s balanced salt solution containing 0.38% sodium citrate, 1 mM adenosine, 2 mM theophylline, and 5% FBS. Dissociated cells were cultured in Iscove’s modified Dulbecco’s medium with L-glutamine, 25 mM HEPES, 5% FBS, 100 units/ml penicillin, 100 μg/ml streptomycin, 25 ng/ml stem cell factor and TPO. Mature Mks were purified on a 1.5–3% BSA gradient and cultured in Cellgro stem cell growth media (CellGenix) for up to 16 h. For immunocytochemistry, Mks were cultured on 200 μg/ml human fibrinogen-coated coverslips for 6 h, fixed for 30 min in 4% paraformaldehyde in PBS, and permeabilized for 15 min with 0.5% TritonX-100 in PBS. Two male animals at the age of nine weeks were used for each genotype group, experimental replicates were biological.

### Whole blood cell and platelet counts

Following dilution of 20 μl of blood with 30 μl acid-citrate-dextrose (ACD) buffer (85 mM trisodium citrate, 65 mM citric acid, 100 mM glucose, pH 5.0) and 50 μl PBS, 1 μl PE Rat Anti-Mouse CD41 (BD Pharmingen, #561850) antibody was added to stain the platelets, and incubated at RT for 30 min. To this, 900 μl of fixative (0.2% formyl saline) was added and incubated for a further 10 min at RT. Finally, 10 μl of this fixed cell suspension was then added to 990 μl PBS. A hemocytometer was used to count whole blood cells using transmitted light, and platelets counted using blue epifluorescence. Four animals at the age of four months and of mixed sex were used for each genotype group, experimental replicates were biological.

### FACS analysis of platelets and leukocytes

Blood was collected by cardiac puncture into 100 μl ACD buffer and mixed with 1 ml DMEM with 10% heparin. To this, 8 ml Erythrocyte-Lysis-Buffer (10 mM KHCO_3_, 155 mM NH_4_Cl, 0.1 mM EDTA, pH 7.5) was added before 15 min incubation on ice. Next, 5 ml PBS/0.5% BSA was added, before centrifugation for 10 min at 200 × *g* at 4 °C, with the resulting cell pellet resuspended in 1 ml PBS/0.5% BSA. In all, 200 μl of cell suspension was used for immunostaining (20 min on ice) to examine immune cell subpopulations (all antibodies from BD Pharmingen, 1:400 dilutions). The cell suspension was washed with 1 ml PBS/0.5% BSA and centrifuged at 600× *g* for 6 min at 4 °C. To detect all immune cells as well as the myeloid fraction, APC-conjugated anti-CD11b (#561690) and APC-conjugated anti-CD45 (#561018) were used. T and B cells were identified by PE anti-CD4 (#557308), PerCP anti-CD8a (#561092), and APC anti-CD19 (#561738) antibodies. Prior to measurement, the cell pellet was resuspended in 300 μl PBS/0.5% BSA, and DAPI was used to exclude dead cells.

To stain platelets, 30 µl ACD/PBS (1:10) with PE rat anti-mouse CD41 (#561850) or PE rat IgG1 control (#551979) antibodies were added to 20 μl blood and incubated for 30 min at RT. 1 ml 0.2% formyl saline and DAPI was then added before FACS analysis. PE rat IgG1 isotype controls were used for gating. For analysis, doublets were gated out on DAPI-negative events. Two female animals at the age of 4 months were used for each genotype group, experimental replicates were biological.

### Platelet isolation

Blood was collected by cardiac puncture into 100 μl ACD buffer. Blood cells were removed by centrifugation at 200 × *g* for 5 min at RT without brake and platelet-rich plasma (PRP) collected. After centrifugation at 200 × *g* for 5 min at RT without brake to remove residual blood cells, the further cleared PRP was collected and centrifuged at 900 × *g* for 10 min at RT without brake. Precipitated platelets were resuspended in ACD and Ca^2+^-free Tyrodes buffer (134 mM NaCl, 2.9 mM KCl, 0.34 mM Na_2_HPO_4_, 12 mM NaHCO_3_, 20 mM HEPES, 1 mM MgCl_2_, 5 mM glucose) and centrifuged at 200 × *g* for 5 min at RT without brake. The further cleared PRP was collected and centrifuged at 900 × *g* for 10 min at RT without brake. The platelet pellet was then resuspended in Ca^2+^-free Tyrode’s buffer for platelet spreading assays.

### Platelet spreading assay

To stimulate activation, 2 mM CaCl_2_ and 0.01 U/ml thrombin (Sigma-Aldrich) were added to 200 μl of rested platelets, and platelets transferred immediately onto fibrinogen-coated coverslips. Following incubation at 37 °C for 45 min, attached platelets were fixed using 10% neutral buffered formalin solution for 20 min. After washing with PBS/0.1% BSA, Alexa Fluor 555 Phalloidin (1:1000 in PBS/0.1% BSA, ThermoFisher Scientific, A34055) was used for staining. Activated platelets were imaged by confocal microscopy (LSM 780, Zeiss), and platelet sizes analyzed using ImageJ^58^. Two female animals at the age of four months were used for each genotype group, experimental replicates were biological.

### Mouse serum generation

BDNF-myc^+/+^;Cre^+/−^ females were crossed with BDNF-myc^+/+^;Cre^−/−^ male mice to generate progeny that were either BDNF-myc^+/+^;Cre^+/−^ or BDNF-myc^+/+^;Cre^−/−^. On 3 consecutive days, dams were sacrificed at E18.5 and blood collected by cardiac puncture. Fetuses were removed, before decapitation to collect blood. Blood was incubated for 1 h at RT, followed by 1 h at 4 °C and centrifugation at 2000 × *g* for 10 min at 4 °C; serum aliquots were stored at −80 °C. Three litters of fetuses were analyzed at E18.5 with a total of 13 BDNF-myc^+/+^;Cre^+/−^pups and 11 BDNF-myc^+/+^;Cre^−/−^ fetuses, experimental replicates were primarily biological. For adult mice, blood was collected by cardiac puncture, and nine Cre^−/−^, six BDNF-myc^+/−^;Cre^+/−^, and five BDNF-myc^+/+^;Cre^+/−^ animals, 2–3 months in age and of mixed sex were analyzed by western blot. Data shown is representative of four experiments, experimental replicates were biological.

### Statistics

RStudio V1.2.5001^59^ was used for graphs and statistical analysis (packages: “car”^60^, “dplyr”^61^, “ggplot2”^62^, “ggpubr”^63^, “pastecs”^64^, and “reshape2”^65^). Median (interquartile range (IQR)) are presented unless otherwise stated. Normality was analyzed by Shapiro–Wilk test, and variance by Levene’s test where appropriate. Group differences were analyzed by Student’s *t* test (two-sided, with unequal variance corrected for as a default parameter; normally distributed data), Wilcoxon signed-rank test (paired data), Wilcoxon rank test (non-paired data), or linear modeling, and associations by Spearman’s rank correlation or linear modeling. Confounding variables controlled for in linear models of human serum values were maternal hypertension^66^, gestational diabetes^35^, maternal BMI at booking^67^, and smoking^41^, alcohol^68^, strenuous exercise^19^, or antidepressant prescription^10^ at any point during pregnancy, determined by previous literature. Analysis of human serum samples used samples collected through the Grown in Wales study^47^. Although this study was not designed to study serum BDNF, it was considered to be sufficiently powered based on a previous study on BDNF levels in human serum which indicated that a cohort size of 60 was required to detect a 20% difference between populations, and 200 for a 10% difference^53^. For analysis of mouse serum samples, no power calculation was performed, as initial data fell into the normal range. Randomization and blinding of mouse samples were not performed, as groups were compared across genotypes.

### Study approval

Full ethical approval for the GiW Study cohort was obtained from the Wales Research Ethics Committee REC reference 15/WA/0004. The research was performed in line with the principles of the Declaration of Helsinki as revised in 2008. Mouse studies were approved by the Cardiff University Ethical Review Board, and all experiments performed within the guidelines of the Home Office Animals (Scientific Procedures) Act, 1986.

## Results

### Higher levels of maternal serum BDNF compared to fetal BDNF

BDNF levels were measured in maternal and fetal serum samples from the GiW cohort^47^. After filtering by the *sample exclusion criteria* (“Methods”), BDNF levels were measured by ELISA in 251 maternal and 212 fetal serum samples (cohort demographics: Table 1).

**Table 1:** Cohort demographic data.

Demographics	Maternal: % (*n*) or median (IQR)	*P*	Fetal: % (*n*) or median (IQR)	*P*
Maternal age at booking	33 (6)	0.223	33.5 (7)	**0.020**
Maternal BMI at booking	26.32 (7.21)	0.645	26.14 (6.84)	0.344
Maternal ethnicity, *% (n)*		–		–
Caucasian	100 (251)		100 (212)	
Fetal sex, *% (n)*		0.498		**2.02e-05**
Female	54 (136)		54 (115)	
Male	46 (115)		46 (97)	
Parity, *% (n)*		0.218		0.374
Multiparous	81 (204)		79 (168)	
Nulliparous	19 (47)		21 (44)	
Gestational age (weeks)	39 (0)	0.159	39 (0)	**0.026**
Smoking at any point in pregnancy, *% (n)*		0.332		0.633
No	89 (224)		89 (189)	
Yes	10 (24)		9 (20)	
Missing	1 (3)		1 (3)	
Alcohol at any point in pregnancy, *% (n)*		0.819		0.330
No	58 (146)		60 (127)	
Yes	40 (100)		38 (81)	
Missing	2 (5)		2 (4)	
Exercise, *% (n)*		0.48		0.502
No	81 (203)		82 (173)	
Yes	18 (46)		17 (36)	
Missing	1 (2)		1 (3)	
Hypertension, *% (n)*		0.735		**0.019**
No	95 (239)		96 (203)	
Yes	4 (9)		3 (7)	
Missing	1 (3)		1 (2)	
Gestational diabetes, % (*n*)		0.073		0.432
No	92 (232)		93 (198)	
Yes	6 (14)		5 (10)	
Missing	2 (5)		2 (4)	
Highest education level, *% (n)*		0.560		0.405
Left before GCSE	5 (13)		7 (14)	
GCSE & vocational	22 (55)		20 (42)	
A-level	12 (31)		11 (24)	
University	29 (74)		30 (64)	
Postgraduate	26 (66)		28 (60)	
Missing	5 (12)		4 (8)	
Family income, *% (n)*		0.064		0.154
<18,000	7 (18)		7 (14)	
18–25,000	10 (24)		8 (16)	
25–43,000	20 (49)		19 (41)	
>43,000	51 (127)		51 (108)	
Do not wish to say	10 (25)		12 (26)	
Missing	3 (8)		3 (7)	
WIMD score	1270 (1183)	0.408	1368 (1158)	0.693
Antidepressants prescribed in pregnancy, *% (n)*		0.198		0.535
No	91 (228)		93 (197)	
Yes	9 (23)		7 (15)	
EPDS score	7 (6)	0.733	7 (6)	0.394
Missing, *n*	7		6	
STAI score	33.5 (12)	0.078	33.5 (13)	0.437
Missing, *n*	31		20	

*EPDS* Edinburgh Postnatal Depression Scale, *STAI* State-Trait Anxiety Inventory, *WIMD* Welsh Index of Multiple Deprivation. For WIMD scores, please see gov.wales/welsh-index-multiple-deprivation. Low scores indicate high deprivation, whilst high scores indicate low levels of deprivation. *P* values indicate the significance of the relationship between serum BDNF levels and the corresponding variable. Bold values indicate *P* < 0.05.

**Fig. 1:**
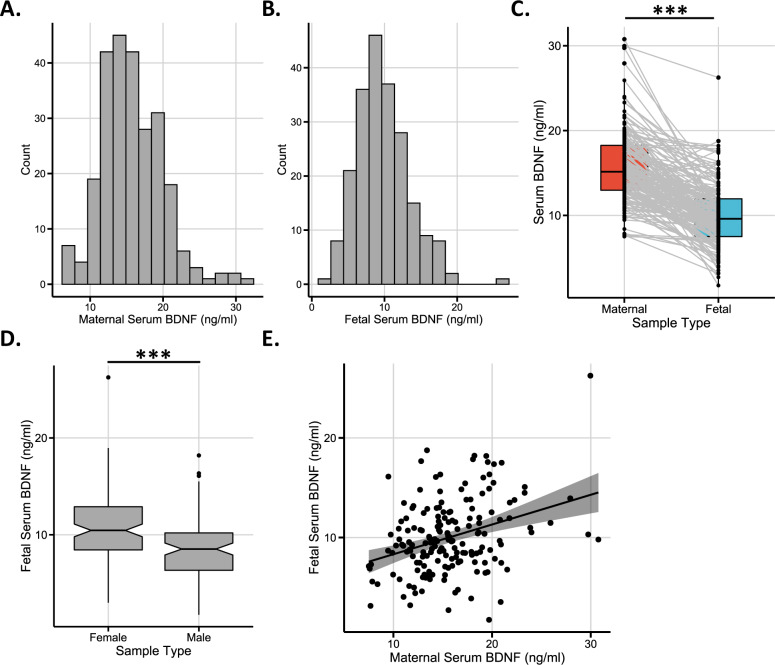
Characterization of serum BDNF levels in maternal and fetal serum. Distribution of serum BDNF values in maternal **A** and fetal **B** samples. **C** Comparison of serum BDNF values between matched maternal and fetal samples (Wilcoxon signed-rank test, *P* < 2.20e-16, *r* = −0.575, *n* = 173 maternal–fetal pairs). Gray lines connect maternal–fetal pairs. **D** Fetal serum BDNF comparison between female and male infant cord blood serum. ***P < 0.001 between female (*n* = 115) and male (*n* = 97) serum samples. After controlling for potential confounding variables (hypertension, gestational diabetes, maternal BMI at booking, smoking, or alcohol at any point in pregnancy, strenuous exercise and prescription of antidepressants) in a linear model, male fetal sex was associated with a decrease in serum BDNF of 2.20 ng/ml (95% confidence intervals (CI): −3.21, −1.19, *P* = 2.63e-5) compared to females (median (IQR)—female: 10.45 (4.45); male: 8.53 (3.86) ng/ml). Boxplot values indicate the following: Box boundaries, 25% and 75% quartiles; mid-line, median; notch, 1.58 × interquartile range (IQR)/sqrt (*n*); whiskers, highest and lowest values within 1.5 × IQR of the box boundary; outliers, values outside the whiskers. Unadjusted vs. adjusted model shown in Supplementary Table 2. **E** Relationship between paired maternal and fetal serum BDNF levels (*r* = 0.290, *P* = 1.09e-04, *n* = 173 pairs, Spearman’s rank correlation; 95% confidence intervals are shaded in gray). In a linear model analyzing this relationship whilst controlling for potential confounding variables (see “Methods”), a 1 ng/ml increase in maternal serum BDNF corresponded to a 0.385 ng/ml increase in fetal serum BDNF (95% CI: 0.244, 0.526, *P* = 2.62e-07).

BDNF values were not normally distributed in maternal (Fig. 1A; *P* = 5.52e-05) or fetal (Fig. 1B; *P* = 1.77e-04) serum. Values were significantly lower in fetal compared to maternal circulation (Fig. 1C; median (IQR)— maternal: 15.15 (5.28); fetal: 9.6 (4.46) ng/ml; *P* < 2.20e−16 from 173 pairs, *r* = −0.575) and lower in male compared to female cord blood (Fig. 1D**;** Supplementary Table 2), similar to previous studies^26,35,41,45^. Fetal sex had no overall effect on maternal serum BDNF values. Mothers with female infants had serum BDNF levels of 15.47 ng/ml (4.88); male infants: 15.2 ng/ml (6.12). Analysis of the relationship between individual maternal and fetal serum samples (Fig. 1E) showed a moderate correlation (Spearman’s rank correlation; *r* = 0.290, *P* = 1.09e−04, *n* = 173 pairs).

### No association between BDNF and maternal EPDS scores

**Fig. 2:**
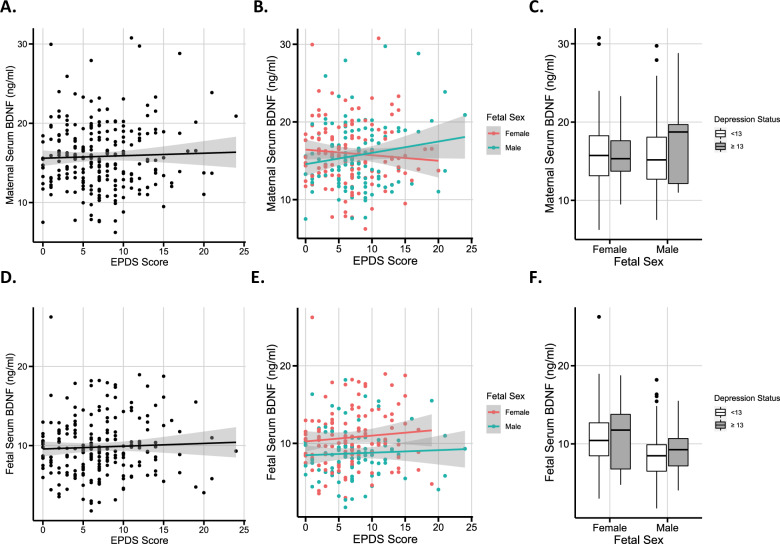
No correlation between serum BDNF levels and maternally reported depression symptoms. **A**, **B** Maternal serum BDNF values plotted against EPDS scores, using (**A**) the whole population (*r* = −0.022, *P* = 0.733, *n* = 244), and (**B**) split by fetal sex (female: *r* = −0.1, *P* = 0.258, *n* = 131; male: *r* = 0.06, *P* = 0.522, *n* = 113, Spearman’s rank correlation). **C** Comparison of maternal serum BDNF values across depression categories and separated by fetal sex (*n*: female infants, EPDS < 13: 112; ≥13: 19; male infants, EPDS < 13: 98; ≥13: 15). **D**, **E** Fetal serum BDNF values plotted against EPDS scores, using (**D**) the whole population (*r* = 0.060, *P* = 0.394, *n* = 206), and (**E**) split by fetal sex (female: *r* = 0.13, *P* = 0.176, *n* = 111; male: *r* = 0.05, *P* = 0.660, *n* = 95, Spearman’s rank correlation). **F** Comparison of fetal serum BDNF values across depression categories and separated by fetal sex (*n*: female infants, EPDS < 13: 94; ≥13: 17; male infants, EPDS < 13: 84; ≥13: 11). Note: fewer samples were available for the fetal serum analysis. EPDS: Edinburgh postnatal depression score. 95% confidence intervals are shaded in gray. Boxplot values indicate the following: Box boundaries, 25% and 75% quartiles; mid-line, median; whiskers, highest and lowest values within 1.5 × IQR of the box boundary; outliers, values outside the whiskers.

Maternal BDNF levels were not correlated with EPDS scores reported at term when all samples were analyzed (Fig. 2A; *r* = −0.022, *P* = 0.733, *n* = 244) or when samples were analyzed by fetal sex (Fig. 2B; male infants: *r* = 0.06; *P* = 0.522; *n* = 113. Female infants: *r* = −0.1; *P* = 0.258; *n* = 131). There was no relationship between maternal BDNF and categorically defined depression using the EPDS ≥ 13 cutoff^50^ (Fig. 2C). There was also no association between fetal BDNF and EPDS scores (Fig. 2D–F).

### Significant association between maternal BDNF and maternal STAI scores

**Fig. 3:**
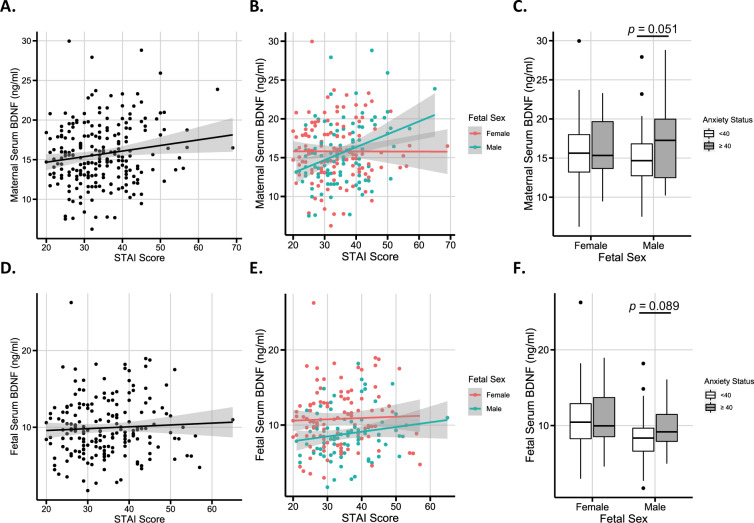
Maternal serum BDNF values correlate with STAI scores as a measure of anxiety. **A**, **B** Maternal serum BDNF values plotted against STAI scores, using (**A**) the whole population (*r* = 0.119, *P* = 0.078, *n* = 220), and (**B**) split by fetal sex (female: *r* = −0.014, *P* = 0.882, *n* = 121; male: *r* = 0.281, *P* = 0.005, *n* = 99, Spearman’s rank correlation). Using a linear model to control for confounding variables and a mean-centered STAI score, a unit increase in STAI score in mothers of males was associated with a 0.185 ng/ml increase in maternal serum BDNF (95% CI: 0.059, 0.309; *P* = 0.004), whilst there was no effect on maternal serum BDNF from either fetal sex or STAI scores alone (*P* = 0.555 and 0.534, respectively). Unadjusted vs. adjusted model shown in Supplementary Table 3. **C** Comparison of maternal serum BDNF values across anxiety categories and separated by fetal sex (*n*: female infants, STAI < 40: 92, ≥40: 29; male infants, STAI < 40: 68, ≥40: 31). **D**, **E** Fetal serum BDNF values plotted against STAI scores, using (**D**) the whole population (*r* = 0.056, *P* = 0.437, *n* = 192), and (**E**) split by fetal sex (female: *r* = 0.073, *P* = 0.458, *n* = 105; male: *r* = 0.131, *P* = 0.226, *n* = 87, Spearman’s rank correlation). **F** Comparison of fetal serum BDNF values across anxiety categories and separated by fetal sex (*n*: female infants, STAI < 40: 79; ≥40: 26; male infants, STAI < 40: 62; ≥40: 25). STAI: Spielberger State-Trait Anxiety Inventory. 95% confidence intervals are shaded in gray. Boxplot values indicate the following: Box boundaries, 25% and 75% quartiles; mid-line, median; whiskers, highest and lowest values within 1.5 × IQR of the box boundary; outliers, values outside the whiskers.

Maternal BDNF levels were not correlated with STAI scores when all samples were analyzed (Fig. 3A; *r* = 0.119; *P* = 0.078; *n* = 220). However, when analyzed by fetal sex STAI scores were significantly associated with maternal serum BDNF in mothers of male (Fig. 3B; *r* = 0.281; *P* = 0.005; *n* = 99; Supplementary Table 3) but not female infants (Fig. 3B; *r* = −0.014; *P* = 0.882; *n* = 121). When analyzed categorically using STAI ≥ 40 suggesting anxiety^51^, BDNF levels between groups were not significantly different (Fig. 3C).

Fetal serum BDNF levels were not correlated with maternal STAI scores when all data were analyzed (Fig. 3D; *r* = 0.056; *P* = 0.437; *n* = 192), or by fetal sex (Fig. 3E; male: *r* = 0.131; *P* = 0.226; *n* = 87. Female: *r* = 0.073; *P* = 0.458; *n* = 105), or by group (Fig. 3F).

### Maternal BDNF does not cross the placenta

**Fig. 4:**
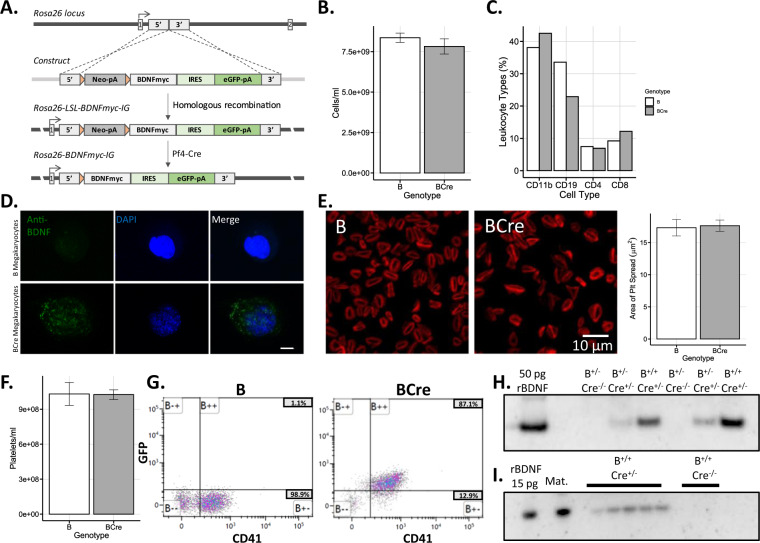
Evidence that BDNF does not transfer across the placenta. **A** Schematic of the generation of the BCre mouse model expressing BDNF from megakaryocytes. **B** Whole blood cell counts (including red blood cells and leukocytes) in BCre and B animals (*t* test: *P* = 0.377; *n* = 4 of each genotype; mean ± SEM). **C** The composition of the major subtypes of leukocytes in BCre and B animals; *n* = 2 animals of each genotype. **D** Immunocytochemical analysis of BDNF in megakaryocytes of BDNF-myc^+/−^;Cre^+/−^ (BCre) and BDNF-myc^+/−^;Cre^−/−^ (B) animals, scale bar = 10 µm. **E** Activation-induced platelet spreading in platelets from BCre and B mice (*t* test: *P* = 0.836; *n* = 20 of each genotype from two animals; mean ± SEM). **F** Blood platelet counts in BDNF-myc^+/+^;Cre^+/−^ (BCre) and BDNF-myc^+/−^;Cre^−/−^ (B) groups (*t* test *P* = 0.960; *n* = 4 of each genotype; mean ± SEM). **G** Expression of GFP in platelets from BCre and B animals by FACS analysis. **H** Western blot analysis of serum BDNF in the BCre mouse model. B BDNF-myc allele, Cre transgenic Cre driver. **I** Western blot analysis of BDNF in the serum of B and BCre fetuses and the corresponding maternal sample. Mat: maternal serum, rBDNF: recombinant BDNF. Western blot is representative of three blots. Three litters of fetuses were analyzed at E18.5 with a total of 13 BDNF-myc^+/+^;Cre^+/−^ pups (nine male/four female) and 11 BDNF-myc^+/+^;Cre^−/−^ pups (six male/five female). Bar graphs show mean values (± standard error of the mean where shown).

To assess the potential for MK-derived BDNF to cross the placenta, we exploited the fact that wild-type mice do not have BDNF present in their blood^69^ and engineered a novel mouse model in which BDNF was expressed from MKs. We inserted a myc-tagged *BDNF-IRES-eGFP* into the *Rosa26* locus (Fig. 4A). When this line is crossed to mice expressing PF4-Cre, driving Cre expression specifically within MKs^57^, mice should express BDNF from MKs (Fig. 4A) as in humans^9^. Through breeding, we generated animals of the following genotypes: BDNF-myc^+/+^;Cre^+/−^ and BDNF-myc^+/+^;Cre^−/−^ (homozygous for the BDNF-myc allele, positive or negative for expression of PF4-Cre), BDNF-myc^+/−^;Cre^+/−^ and BDNF-myc^+/−^;Cre^−/−^ (heterozygous for BDNF-myc, positive or negative for PF4-Cre) and lastly BDNF-myc^−/−^;Cre^+/−^ and BDNF-myc^−/−^;Cre^−/−^ (wild-type in the BDNF-myc allele, positive or negative for PF4-Cre). All genotypes were viable and fertile, with no overt phenotype. The hematological profile of mice carrying one or two alleles of BDNF-myc and the Cre transgene (BCre; recombined scenario) was similar to mice carrying a BDNF-myc allele in the absence of the Cre transgene (unrecombined) (B) (Fig. 4B, C). In animals carrying the Cre transgene and one or two alleles of the BDNF-myc (BCre: BDNF-myc^+/+^;Cre^+/−^ or BDNF-myc^+/−^;Cre^+/−^), BDNF could be detected in MKs (Fig. 4D). To reach the bloodstream, MK-expressed BDNF must be packaged into platelets, from which it can be released during platelet activation. Activation-induced platelet spreading (Fig. 4E) and platelet counts (Fig. 4F) were similar between BCre and B mice. In animals without the Cre transgene (B), ~0.5% of white blood cells (0.52 ± 0.1% (SD)) were positive for eGFP whereas over 85% of platelets from BCre mice expressed eGFP (Fig. 4G). BDNF-myc was detectable by western blot in the serum of BCre mice and not detectable in mice without the Cre transgene (Fig. 4H and Supplementary Fig. 1). By ELISA, the mean (± standard error of the mean) serum BDNF values in young adult mice (12 weeks old) were 10.6 ± 1.45 ng/ml in BDNF-myc^+/−^;Cre^+/−^ animals (*n* = 4; three female, one male), and 32.3 ± 3.70 ng/ml in BDNF-myc^+/+^;Cre^+/−^ animals (*n* = 3; three female). Thus, in this model MK-derived BDNF is present in serum at levels comparable to those determined in adult human serum^53^.

Having confirmed MK expression of BDNF, this model was used to test if MK-derived BDNF in maternal blood could cross the placenta. BDNF-myc^+/+^;Cre^+/−^ females expressing MK-derived BDNF were crossed with BDNF-myc^+/+^;Cre^−/−^ male mice to generate fetuses homozygous for the BDNF-myc insertion, with approximately half lacking the Cre transgene and thus intrinsic serum BDNF. Cre-negative fetuses (BDNF-myc^+/+^;Cre^−/−^) could only have serum BDNF if this were maternally-derived. Maternal and fetal blood were collected at E18.5 from 3 litters, serum generated and BDNF expression analyzed by western blot. BDNF-myc was detectable in maternal serum and serum from BDNF-myc^+/+^;Cre^+/−^ fetuses but absent in serum from the BDNF-myc^+/+^;Cre^−/−^ fetuses (Fig. 4I).

## Discussion

This is the first study to interrogate the relationship between maternal and fetal serum BDNF at term in the context of maternal symptoms of depression and anxiety. We discovered that maternal symptoms of anxiety correlated with significantly raised maternal serum BDNF exclusively in mothers of boys. We did not find similarly raised BDNF levels in the male infants, in line with the results obtained with our new animal model in which we found no evidence that MK-derived BDNF, the main source of BDNF in humans, crosses the placenta.

Elevated maternal BDNF was specifically associated with maternally reported anxiety symptoms and not symptoms of depression. Symptoms of depression have been associated with lower serum BDNF in non-pregnant adults^10,11^. Reports of associations between anxiety symptoms and serum BDNF have been mixed, with one systematic review concluding lower BDNF only in association with obsessive-compulsive disorder^70^. Animal studies suggest that overexpression of BDNF in the brain can simultaneously improve performance in tests related to depression while facilitating anxiety-like behavior^71^. This would be essentially consistent with our findings of a contrasting relationship of serum BDNF with anxiety and depressive symptoms. Testing the relationship between MK-derived BDNF and behavioral outcomes will now be possible using the animal model described here.

Our finding of elevated maternal BDNF was exclusive to the mothers of boys reporting higher anxiety symptoms. We currently do not know whether this is a causal relationship nor, if causal, the direction of causality. Anxiety symptoms, or factors associated with anxiety, may drive changes in maternal BDNF that are only permissible in those mothers with a male fetus. Alternatively, under conditions of maternal anxiety, the male fetus or placenta may produce signals driving elevations in maternal BDNF. A third possibility is that elevated BDNF is causing anxiety only in mothers with boys. Subsequent studies with our new mouse model should help tease apart these complex relationships. In addition to determining causality, it will also be important to establish whether changes in maternal BDNF occur as a result of alterations in MK proliferation, MK differentiation, BDNF expression within MKs, and/or platelet release of BDNF into the circulation. Another important question to address is the function of MK-derived BDNF in development or disease. While further work is required, our identification of this sex-specific association between maternal anxiety and maternal serum BDNF provides a highly novel example of the influence of fetal sex on the maternal state.

Whether these perturbations in maternal serum BDNF are transmitted to the fetus is an important question, given the known critical function of BDNF in the central nervous system and our increasing understanding of its function in the periphery, including in energy homeostasis and regulation of glucose levels^72,73^. We found no evidence that BDNF can cross the placenta, at least in mice. Maternal blood BDNF is contained within platelets and is not found as free serum protein. Furthermore, the hemochorial placenta provides a physical barrier with three continuous cellular layers between the maternal and fetal blood systems in both mice and humans^22^. Some proteins can cross the placenta by an active process termed receptor-mediated pinocytosis^23^. We were unable to detect TrkB or p75 by western blot analysis of placental extracts or by immunostaining in the labyrinth of the mouse placenta where transport occurs (data not shown) consistent with the lack of BDNF transplacental transfer in our model. While we cannot formally exclude the possibility that there are species differences in the placental transfer of BDNF as observed with the transfer of maternal antibodies^74^, BDNF receptors do not appear to be expressed in the mature human placenta^75^.

An important feature of our study is the number of paired maternal:fetal BDNF samples measured at term. However, we only measured mood symptoms once during pregnancy. We do not know when anxiety symptoms first manifested but the persistence of symptoms after birth^47^ and the nature of the questionnaire, measuring trait not state anxiety, suggests prolonged symptomology. Our cohort is relatively homogeneous and it will be important to replicate these findings in more diverse cohorts, and across other modes of delivery.

In summary, we have discovered a sex-specific association between maternally reported symptoms of anxiety and elevated maternal serum BDNF at term, specifically in pregnancies with male infants. We find no evidence that maternal serum BDNF crosses the placenta either from our human study or in our new humanized mouse model. We conclude that the placenta provides a barrier protecting the fetus against potentially damaging fluctuations in maternal BDNF that occur under conditions of stress.

## Supplementary information

Supplemental Material
