# Supplementary material for: The placenta protects the fetal circulation from anxiety-driven elevations in maternal serum levels of brain-derived neurotrophic factor
Source: Transl Psychiatry. 2021 Jan 18;11:62. doi: 10.1038/s41398-020-01176-8 (PMC7813890; doi:10.1038/s41398-020-01176-8)
Supplement: Supplementary file 1 — Supplemental Material [file 41398_2020_1176_MOESM1_ESM.docx]

**Supplementary Materials and Methods**

*BDNF immunoprecipitation from serum*

250 μl serum pooled from 2 BDNF^+/-^;Cre^-/-^ animals (B) and 5 BDNF^+/-^;Cre^+/-^ (BCre) animals (mixed sex, 14-16 months old) was incubated with 2.8 μg biotin-BDNF-Ab#1 overnight at 4 °C. The following day, 500 μl PBS and 50 μl washed Dynabeads M-280 Streptavidin (ThermoFisher Scientific), were added and incubated for 1 hr at room temperature. Dynabeads were washed three times with 0.3% Triton X-100 in PBS prior to use. 50 μl RIPA buffer, 5 μl DTT, and 12.5 μl 4 x LDS loading buffer were added, before samples were heated at 90 °C for 5 min and 13.5 μl sample loaded onto SDS-PAGE gels.

*Western Blots*

Different amounts of recombinant BDNF-myc protein were loaded onto SDS-PAGE gels as standards. Membranes were incubated with 1 μg/ml anti-BDNF antibody 3C11hIgGLuc2 (a gift from Icosagen) or 1 μg/ml anti myc-tag antibody (Cell Signaling Technology, clone 71D10). For the 3C11hIgGLuc2 antibody, no secondary antibody was used and the blot was developed with Nano-Glo Luciferase Assay System (Promega, #1110) according to the manufacturer’s instructions. For the 71D10 antibody, the standard protocol as described in the main methods section was used for detection, using the secondary antibody HRP-conjugated anti-Rabbit IgG (Promega, #W4011). BDNF-myc was quantified by 3C11hIgGLuc2 antibody using ImageJ software^1^.

**Supplementary Table 1:** **Previous literature reporting on serum BDNF levels during the perinatal period.** Search terms: Pregnancy BDNF (filter: humans) and studies found therein. Studies excluded when it is not specifically serum measured, or there is no clear indication of serum vs. plasma. Restricted to studies including full-term infants and their mothers in the immediate perinatal period. Only studies with a manuscript available in English were included. Abbreviations: AD: Anxiety disorders; AGA: Appropriate for gestational age; BDI: Beck Depression Inventory; BDNF: Brain-derived neurotrophic factor; CC: Crack cocaine; CI: Confidence intervals; EPDS: Edinburgh Postnatal Depression Score; GAD: generalized anxiety disorder; GDM: Gestational diabetes mellitus; Gest: gestational; HC: Healthy control; IDA: Mothers with iron deficiency anemia; IQR: Inter-quartile range; IUGR: Intra-uterine growth restriction; Mat.: Maternal; MDD: Major depressive disorder; PPD: Post-partum depression; SD: Standard deviation; SEM: Standard error of the mean; trim: Trimester; UC: Umbilical cord. Studies are ordered by year, then alphabetically by first author.

| **Paper** | **Sample No. & Type** | **Sample Collection &**  **Preparation** | **Average Serum BDNF Values (ng/ml)** | **Findings Relevant to Current Study** |
| --- | --- | --- | --- | --- |
| **Chouthai *et al.*, 2003**^2^ | 60 UC blood samples from different gest. ages. | From UC into a syringe, centrifuged, and stored at -80 °C. | Mean (SD):  UC from 24-28 week infants: 0.88 (0.39).  UC from 29-35 week infants: 1.4 (0.62).  UC from 36 weeks and over infants: 2.2 (0.36). | Increase in serum BDNF with gest. age.  A trend for increased serum BDNF was seen in female compared to male infants (mean ± SD: 1.6 ± 0.65 vs 1.4 ± 0.7 ng/ml respectively), this was not significant. Antenatal steroids increased UC serum BDNF. |
| **Malamitsi-Puchner *et al.,* 2004**^3^ | 30 full term and 15 preterm maternal and UC blood samples. | Maternal: At 1^st^ stage of labor. UC: From doubly clamped UC at delivery, into pyrogen-free tubes, immediately centrifuged and stored at -80 °C. | Median (range):  Full-term Mat.: 5.4 (2.8-12.7).  Pre-term Mat.: 6.5 (4.8-9.1).  Full-term infants: 2.5 (1.2-13.3).  Pre-term infants: 1.85 (1.4-2.1). | Mat. serum was higher than infant serum, no difference by gest. age.  Full term infants had higher UC serum BDNF than preterm infants. No effect of infant sex. |
| **Lommatzsch *et al.*, 2006**^4^ | 40 pregnant women and 40 non-pregnant controls. | Several time points, including 37 weeks. Between 1500-1900 h, into additive-free tubes, kept on ice for 60 min and centrifuged for 10 min, 2000 x *g* at 4 °C, stored at -80 °C. | Median (range):  Non-pregnant women: 12.6 (1.2-47.8).  Pregnant women at 37 weeks: 2.9 (0.4 – 33.1). | Serum BDNF lower during pregnancy than in non-pregnant women. Reported trend towards a negative correlation between EPDS scores and serum BDNF but not significant (*r* = -0.02, *p*>0.05). |
| **Cannon *et al.*, 2008**^5^ | 444 maternal samples, 252 UC samples. | Maternal blood samples were collected during pregnancy, infant UC samples at delivery.  Stored at -20 °C for 45-50 years. | Mean (SEM):  Mothers: approx. 0.4 (0.06).  UC: approx. 0.43 (0.13). | Premature infants had lower BDNF levels (0.37 ± 0.024) than full term infants (0.44 ± 0.016). Hypoxia had contrasting effects on BDNF levels from UC blood when split by future psychoses diagnoses (schizophrenia and affective psychosis). |
| **Spulber *et al.*, 2010**^6^ | 395 UC blood samples. | From UC immediately following delivery, serum isolated and kept frozen. | Mean (SEM):  UC: 9.3 (4.9).  UC from male infants only: 8.6 (4.4).  UC from female infants only: 10.1 (5.3). | Serum BDNF higher in female infants than males. BDNF in UC blood increases with gest. age.  Negative correlation between prenatal MeHG exposure and serum BDNF only in females born to non-smoking mothers. Smoking increased UC serum BDNF in female infants only. |
| **Uguz *et al.*, 2013**^7^ | 44 UC blood samples. | During C-section, blood taken from UC, immediately centrifuged for 10 min, 300 x *g* at 4 °C, stored at -80 °C. | Mean (SD):  Infants from mothers without GAD: 2.08 (0.9).  Infants from mothers with GAD: 1.03 (0.4). | Infants of women with GAD had significantly lower levels of BDNF in UC blood. |
| **Dhiman *et al.*, 2014**^8^ | Maternal serum from 103 women. | 5 ml taken at assessment (24-48 h after delivery). | Mothers with PPD: 199 (74).  Mothers without PPD: 259 (119). | Lower levels of serum BDNF found in mothers with PPD. |
| **Garces *et al*., 2014**^9^ | Serum from 58 women (16 non-pregnant, 42 pregnant). | Blood taken in the 3 different trimesters, after overnight fast. Serum separated by centrifugation and stored at -80 °C. | Mean (SD):  Non pregnant controls: 31.7 (8.1).  1^st^ trim: 19.4 (7.5).  2^nd^ trim: 24 (9.5).  3^rd^ trim: 25.1 (8.2). | BDNF decreased early in pregnancy but increased over the trimesters. Negative correlation between serum levels of BDNF and fasting levels of glucose. |
| **Flöck *et al.*, 2016**^10^ | Maternal and UC blood samples from 108 newborn/maternal pairs. | Blood taken from mothers at admission to labor ward; taken from UC after clamping. To 4 °C until centrifuged for 10 min at 4000 rpm, stored at -70 °C. | Median (IQR):  UC blood: 2.6 (2.0).  Maternal: 3.1 (2.4). | Mat. serum was higher than infant serum. UC BDNF increases with gest. age. Correlation of *r* = 0.251 between UC blood and mat. serum BDNF levels. No difference between male (2.34 ± 2.0 ng/ml) and female (2.9 ± 2.0 ng/ml) UC serum BDNF. |
| **Gao *et al.*, 2016**^11^ | Samples from 340 mothers. | Fasting blood taken into Rapid Serum Tube 24-48 h post-delivery (0700-0800 h), centrifuged and stored at -80 °C. | Median (IQR):  Mothers with PPD: 8.8 (6.7-11.5).  Mothers w/o PPD: 14.3 (11.4-17.5). | Relationship between reduced serum BDNF levels at admission and development of PPD levels within 3 months, with a negative correlation between serum BDNF levels and EPDS scores at this 3-month assessment (*r* = -0.248). Stressful life events also associated with lower serum BDNF levels. |
| **Hodyl *et al.*, 2016**^12^ | UC from 119 late pre-term and 129 full-term infants. | Cord blood serum samples obtained from infants. | Median (IQR):  UC from late preterm infants, no steroids: 1.7 (0.9-2.3).  UC from late preterm infants, steroids: 1.4 (0.9 – 2.3).  Term: 1.3 (0.9-1.9). | No increase in BDNF with gest. age and antenatal steroid use. |
| **Wang *et al*., 2016**^13^ | 149 UC blood samples. | Umbilical cord blood collected after delivery, allowed to clot, centrifuged for 1500 rpm for 20 min, stored at -80 °C. | Not stated. | Negative correlation between mat. cadmium exposure and both UC BDNF levels and Gesell Developmental Schedules score. |
| **Yu *et al.*, 2016**^14^ | 377 UC blood samples. | UC blood collected, separated and stored at -80 °C. | Ranges from 0 to 40. | UC serum BDNF negatively correlated with Manganese levels. BDNF levels were negatively associated with fine motor scores, and positively associated with personal–social scores as measured by Gesell Developmental Inventory. |
| **Cai *et al.*, 2017**^15^ | 90 UC blood samples. | Cord blood taken immediately after delivery, centrifuged at 1800 rpm for 30 min, stored at -80 °C. | Mean (SE):  Macrosomic infants: 204 (14.3).  Non-macrosomic infants: 258.9 (14.1). | UC BDNF increases with gest. age in non-macrosomic infants.  UC BDNF lower in macrosomic infants than in non-macrosomic infants. |
| **Mardini *et al.*, 2017**^16^ | 156 UC blood samples and maternal blood. | Blood drawn in the morning and kept refrigerated until centrifugation (within 2 h). Centrifuged at 4000 x *g* at 4 °C and stored at -80 °C. | Adjusted mean (95% CI):  CC-exposed infants: 3.9 (2.3-5.4).  CC-non-exposed infants: 0.85 (0.5-1.2).  CC-exposed mothers: 4.0 (2.9-5.2).  CC-non-exposed mothers: 6.7 (5.6-7.7). | UC BDNF higher in infants exposed to CC *in utero*. Serum BDNF lower in mothers exposed to CC. |
| **Akbaba *et al.*, 2018**^17^ | 136 UC blood samples. | Blood obtained from UC, immediately centrifuged at 300 x *g*, 4 °C for 10 min and stored at -80 °C. | Mean (SD):  Infants from HC mothers: 229.0 (73.4).  Infants from mothers with MDD: 193.6 (65.0).  Infants from mothers with AD: 215.0 (70.8). | No difference in UC serum BDNF between infants from HC, MDD, or AD mothers. |
| **Basu *et al.*, 2018**^18^ | 90 UC blood samples. | Free flowing cord blood collected, serum stored at -20 °C. | Mean (SD):  Infants from mothers with IDA: 7.4 (4.2).  Infants from mothers without IDA: 15.3 (1.2). | UC serum BDNF lower in infants born to mothers with anemia, with a significant decline reported with the severity of anemia. |
| **Briana *et al.*, 2018**^19^ | 80 UC blood samples. | Mixed arteriovenous samples collected immediately after birth by puncture of double-clamped UC, into pyrogen-free tubes, immediately centrifuged, and stored at -80 °C. | Mean (SD):  UC of AGA infants from mothers without GDM: 10.7 (6.1).  UC of AGA infants from mothers with GDM: 9.1 (3.9).  UC of IUGR infants from mothers with GDM: 8.1 (3.7).  UC from LGA infants from mothers with GDM: 6.7 (2.3). | Fetal BDNF lower in diabetic pregnancies. UC serum BDNF was higher in female infants. |
| **Ferrari *et***  ***al.*, 2018**^20^ | Maternal serum from 34 women. | Fasted blood taken at 36 weeks into serum venipuncture tubes and clotted over 30 min. Centrifuged for 10 min, 4000 x *g* at 4 °C and stored at -20 °C. | Mean (SEM):  Mothers on exercise program: 6.5 (0.5).  Control mothers: 3.4 (0.4). | Mat. serum BDNF increased in mothers who undertook an exercise program. |
| **Yusrawati *et al.*, 2018**^21^ | 40 UC blood samples. | UC blood taken immediately after birth. | Mean (SD):  Infants from mothers with normal ferritin: 3.8 (1.4).  Infants from mothers with low ferritin:  2.8 (1.2). | UC serum BDNF lower in infants born to mothers with low ferritin. |
| **Pawluski *et al.*, 2019**^22^ | 55-63 maternal samples at delivery. | Collected up to 2 h after delivery. Centrifuged at 1300 x *g* for 8 min at 4 °C, stored at -80 °C. | Mean (SEM):  SSRI-treated women: 17.2 (0.9).  Non SSRI-treated 16.6 (0.9). | Lower BDNF levels at delivery were associated with increased depressive symptoms (not significant), but this trend was not seen in SSRI-treated women. |
| **Sonmez *et al.***, **2019**^23^ | 48 UC blood samples. | Blood obtained from umbilical vein during caesarean section, centrifuged for 10 min, 3000 x *g* at 4 °C, stored at -80 °C. | Mean (SD):  Infants from mothers with depression diagnosis: 1.0 (0.5).  Infants from mothers with no depression diagnosis: 2.1 (0.9). | UC serum BDNF lower in infants from mothers diagnosed with depression, though no correlation with BDI scores. |

**Supplementary Table 2. Male fetal serum BDNF is significantly lower than female fetal serum BDNF.** Multiple linear regression models assessing the association between fetal sex and fetal serum BDNF (ng/ml). Adjusted model is adjusted for maternal hypertension, gestational diabetes, maternal BMI at booking, and smoking, alcohol, strenuous exercise or antidepressant prescription at any point during pregnancy. BDNF: Brain-derived neurotrophic factor; CI: Confidence intervals.

| **Model** | **Fetal Sex** | ***P*** | **B** | **95% CI** |
| --- | --- | --- | --- | --- |
| Unadjusted | Female | *ref* |  |  |
|  | Male | 3.02e-5 | -2.10 | -3.07, -1.13 |
|  | | | | |
| Adjusted | Female | *ref* |  |  |
|  | Male | 2.63e-5 | -2.20 | -3.21, -1.19 |

**Supplementary Table 3:** **Maternal serum BDNF is associated with levels of anxiety in mothers with male infants.** Multiple linear regression models assessing the association between maternal anxiety levels (using mean-adjusted STAI scores), and maternal serum BDNF (ng/ml) in mothers of male and female infants. Adjusted model is adjusted for maternal hypertension, gestational diabetes, maternal BMI at booking, and smoking, alcohol, strenuous exercise or antidepressant prescription at any point during pregnancy. BDNF: Brain-derived neurotrophic factor; CI: Confidence intervals; STAI: State-Trait Anxiety Inventory.

| **Model** | **Factor** | ***P*** | **B** | **95% CI** |
| --- | --- | --- | --- | --- |
| Unadjusted | Maternal Anxiety | 0.983 | -0.001 | -0.080, 0.078 |
|  | Fetal Sex | 0.487 | -0.366 | -1.405, 0.672 |
|  | Maternal Anxiety:  Male Infants | **0.007** | 0.167 | 0.047, 0.286 |
|  | | | | |
| Adjusted | Maternal Anxiety | 0.534 | -0.026 | -0.110, 0.057 |
|  | Fetal Sex | 0.555 | -0.323 | -1.399, 0.753 |
|  | Maternal Anxiety:  Male Infants | **0.004** | 0.185 | 0.060, 0.309 |

**Supplementary Figure 1:** **Western blot analysis of serum immunoprecipitated (IP) BDNF-myc, detected with anti-BDNF and anti-myc antibodies.** Different amounts of recombinant BDNF-myc protein were also loaded onto SDS-PAGE as standards. Membranes were incubated with 1 μg/ml anti-BDNF antibody 3C11hIgGLuc2 (a gift from Icosagen) or 1 μg/ml anti myc-tag antibody (Cell Signaling Technology, 71D10). While the anti-BDNF antibody 3C11hIgGLuc2 can detect BDNF at pg levels, the myc antibody 71D10 can only detect ng of protein (**A**). Based on the standard curve generated from the 3C11hIgGLuc2 antibody (**B**), the BCre sample contained 640 pg BDNF-myc (IP from 50 μl serum), which is at the lower end for myc antibody detection (see a faint band in Fig **A** lower panel). The availability of anti-BDNF antibody 3C11 and its derivatives, such as 3C11hIgGLuc2 (which is a fusion with Luciferase), significantly simplified detection of BDNF because of their superior sensitivity relative to any previous tested BDNF antibodies, and any myc-tag antibodies.

| **A.** |
| --- |
| *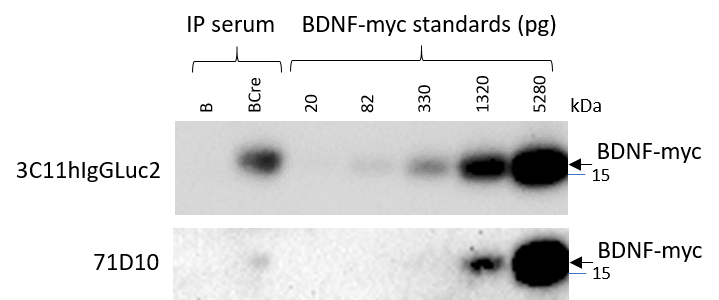* |
| **B.** |
|  |
|  |

**Supplementary References**

1 Schneider C.A., Rasband W.S., Eliceiri K.W. NIH Image to ImageJ: 25 years of image analysis. *Nat. Methods* **9**, 671–675 (2012).

2 Chouthai N.S., Sampers J., Desai N., Smith G.M. Changes in neurotrophin levels in umbilical cord blood from infants with different gestational ages and clinical conditions. *Pediatr. Res.* **53**, 965–969 (2003).

3 Malamitsi-Puchner A., Economou E., Rigopoulou O., Boutsikou T. Perinatal changes of brain-derived neurotrophic factor in pre- and fullterm neonates. *Early Hum. Dev.* **76**, 17–22 (2004).

4 Lommatzsch M. *et al*. Maternal serum concentrations of BDNF and depression in the perinatal period. *Psychoneuroendocrinology* **31**, 388–394 (2006).

5 Cannon T.D., Yolken R., Buka S., Torrey E.F., Collaborative Study Group on the Perinatal Origins of Severe Psychiatric Disorders. Decreased Neurotrophic Response to Birth Hypoxia in the Etiology of Schizophrenia. *Biol. Psychiatry* **64**, 797–802 (2008).

6 Spulber S. *et al.* Effects of maternal smoking and exposure to methylmercury on brain-derived neurotrophic factor concentrations in umbilical cord serum. *Toxicol. Sci.* **117**, 263–269 (2010).

7 Uguz F. *et al.* Maternal generalized anxiety disorder during pregnancy and fetal brain development: A comparative study on cord blood brain-derived neurotrophic factor levels. *J. Psychosom. Res.* **75**, 346–350 (2013).

8 Dhiman P., Say A., Rajendiren S., Kattimani S., Sagili H. Association of foetal APGAR and maternal brain derived neurotropic factor levels in postpartum depression. *Asian J. Psychiatr.* **11**, 82–83 (2014).

9 Garcés M.F. *et al.* Brain-derived neurotrophic factor is expressed in rat and human placenta and its serum levels are similarly regulated throughout pregnancy in both species. *Clin. Endocrinol. (Oxf)* **81**, 141–151 (2014).

10 Flöck A. *et al.* Determinants of brain-derived neurotrophic factor (BDNF) in umbilical cord and maternal serum. *Psychoneuroendocrinology* **63**, 191–197 (2016).

11 Gao X., Wang J., Yao H., Cai Y., Cheng R. Serum BDNF concentration after delivery is associated with development of postpartum depression: A 3-month follow up study. *J. Affect. Disord.* **200**, 25–30 (2016).

12 Hodyl N.A. *et al*. Antenatal steroid exposure in the late preterm period is associated with reduced cord blood neurotrophin-3. *Early Hum. Dev.* **101**, 57–62 (2016).

13 Wang Y. *et al.* Effects of prenatal exposure to cadmium on neurodevelopment of infants in Shandong, China. *Environ. Pollut.* **211,** 67–73 (2016).

14 Yu X. *et al*. The role of cord blood BDNF in infant cognitive impairment induced by low-level prenatal manganese exposure: LW birth cohort, China. *Chemosphere* **163**, 446–451 (2016).

15 Cai Q.Y. *et al.* Placental and cord blood brain derived neurotrophic factor levels are decreased in nondiabetic macrosomia. *Arch. Gynecol. Obstet.* **296**, 205–213 (2017).

16 Mardini V. *et al.* TBARS and BDNF levels in newborns exposed to crack/cocaine during pregnancy: A comparative study. *Braz. J. Psychiatry* **39**, 263–266 (2017).

17 Akbaba N. *et al*. Neurotrophins and neuroinflammation in fetuses exposed to maternal depression and anxiety disorders during pregnancy: a comparative study on cord blood. *Arch. Womens Ment. Health* **21**, 105–111 (2018).

18 Basu S., Kumar D., Anupurba S., Verma A., Kumar A. Effect of maternal iron deficiency anemia on fetal neural development. *J. Perinatol.* **38**, 233–239 (2018).

19 Briana D.D. *et al*. Differential expression of cord blood neurotrophins in gestational diabetes: the impact of fetal growth abnormalities. *J. Matern. Neonatal Med.* **31**, 278–283 (2018).

20 Ferrari N. *et al.* Exercise during pregnancy and its impact on mothers and offspring in humans and mice. *J. Dev. Orig. Health Dis.* **9**, 63–76 (2018).

21 Yusrawati, Rina G., Indrawati L.N., Machmud R. Differences in brain-derived neurotrophic factor between neonates born to mothers with normal and low ferritin. *Asia Pac. J. Clin. Nutr.* **27**, 389–392 (2018).

22 Pawluski J.L., Brain U., Hammond G.L., Oberlander T.F. Selective serotonin reuptake inhibitor effects on neural biomarkers of perinatal depression. *Arch. Womens Ment. Health* **22**, 431–435 (2019).

23 Sonmez E.O. *et al.* Effect of maternal depression on brain-derived neurotrophic factor levels in fetal cord blood. *Clin. Psychopharmacol. Neurosci.* **17**, 308–313 (2019).
